# Early neonatal Glutaric aciduria type I hidden by perinatal asphyxia: a case report

**DOI:** 10.1186/s13052-018-0450-8

**Published:** 2018-01-15

**Authors:** Giacomo Biasucci, Nicola Morelli, Federica Natacci, Massimo Mastrangelo

**Affiliations:** 1 Pediatrics & Neonatology Unit, “Guglielmo da Saliceto” City Hospital, Cantone del Cristo, 50, 29121 Piacenza, Italy; 2 Neurology and Radiology Unit, “Guglielmo da Saliceto” City Hospital, Piacenza, Italy; 3 Medical Genetics Unit, Fondazione IRCCS Cà Granda Ospedale Maggiore Policlinico, Milan, Italy; 4 Pediatric Neurology Unit, “Vittore Buzzi” Children’s Hospital, ASST FBF-Sacco, Milan, Italy

**Keywords:** Perinatal asphyxia, Neonatal Glutaric aciduria type 1, Neonatal epileptic seizures, Post-natal neurological damage, Expanded newborn screening

## Abstract

**Background:**

Perinatal asphyxia (PA) occurs in about 2 to 10 per 1000 live full-term births. Although neonatal epileptic seizures are observed in up to 60% of cases, PA may mimic or subtend other conditions. Hypoxia related brain injury is particularly relevant, as it may have permanent effects on neuropsychomotor development. Antepartum obstetric conditions, may, in turn, lead to hypoxic-ischemic damage to the fetus and the newborn, often underlying PA. Herein, a case of PA that hid and triggered signs and symptoms of Glutaric Aciduria type I (GA-I), is reported.

**Case presentation:**

R.F. was born at term after prolonged labour, by induced vaginal delivery with the Kristeller manoeuvre. He presented with severe asphyxia and asystoly. Immediate cardiopulmonary resuscitation promptly restored cardiorespiratory parameters, allowing for early extubation 30 min after. During the following hours, severe axial muscle hypotonia with an increased tone of the limb extensor muscles became evident. The absence of crying and archaic reflexes persisted and there was an onset of generalized tonic or clonic seizure. First level metabolic and inflammatory markers were within the normal range. An inherited metabolic disease was then suspected, due to the persistent clinical signs of severe neurological damage without any detectable septic parameter. GA-I was assessed and specific treatment started without any clinical improvement, although ensuring adequate growth and metabolic control. Thereafter, the baby developed a severe encephalopathy with drug resistant epileptic seizures. The progression of the neurological damage and a CVC-related sepsis led him to exitus at 2 years.

**Conclusions:**

To the best of our knowledge, this is the first case of early post-natal onset of GA-I reported in literature to date, in the absence of expanded newborn screening (NBS) programme. As expanded NBS programmes for inborn errors of metabolism have not yet been internationally adopted, we are of the opinion that such diseases may well be hidden by misleading signs and symptoms imputable to other more frequent harmful clinical conditions. Moreover, it would be advisable that neonatologists be trained to include GA-I in the differential diagnosis of neurological damage secondary to PA.

## Background

Perinatal asphyxia (PA) occurs in about 2 to 10 per 1000 live full-term births, due to oxygen deprivation during labour of sufficient duration to cause injury, mostly to the brain. Although neonatal epileptic seizures are often observed in up to 60% of cases [1], neonatal asphyxia may mimic or subtend other conditions. Hypoxia may affect almost all the neonate’s organs such as heart, lungs, liver, gut and kidneys. However, brain damage is particularly relevant, as it may have permanent effects on neuropsychomotor development and is of utmost concern as it is manifested as either mental impairment (such as developmental delay or intellectual disability), or physical injury, such as spasticity.

Antepartum risk factors are the most common underlying factors in PA and include maternal hypotension, haemorrhage, placental insufficiency or abruption which may cause hypoxic-ischemic damage to the foetus and/or the newborn.

Herein, a case of PA, where asphyxia both underpinned and hid the triggering signs and symptoms of Glutaric Aciduria type I (GA-I) [2], is described. PA was diagnosed on the basis of the clinical and biochemical findings, before the regional expanded newborn screening (NBS) programme was implemented. To the best of our knowledge, this is the first case of early post-natal onset of GA-I reported in literature so far.

## Case presentation

RF, male, of non consanguineous Caucasian parents, was born at term after prolonged labour, that necessitated oxytocin induced vaginal delivery with the Kristeller manoeuvre. His auxometric parameters, −including head circumference- were adequate for gestational age. Severe asphyxia and asystoly started soon after birth.

Immediate cardiopulmonary resuscitation up to endotracheal intubation with manual ventilation promptly restored cardiorespiratory parameters, allowing for early extubation 30 min later, with subsequent spontaneous breathing. There was no further need for oxygen supply as the cardiopulmonary functions remained stable.

The first arterial hemogasanalysis was performed 20 min after birth while the baby was still intubated and ventilated. He also had severe metabolic acidosis (pH: 7.0, pCO_2_: 24 mmHg, pO_2_: 242 mmHg, HCO_3_^−^: 6 mmol/L, BE: – 23 mmol/L). Bicarbonate correction was started. It was first administered slowly as an intravenous (i.v.) bolus, then continuous i.v. infusion was used over a 24 h period, along with gluco-saline solution, tailored to his daily fluid requirements.

At 30 min after birth, the neonate had good skin perfusion, no clinically evident malformation or dysmorphism, but severe and diffuse hypotonia, absence of crying and archaic reflexes; a heart rate of 120 bpm and a respiratory rate of 40/min.

The baby dropped into a stuporous state with a fixed gaze and epileptic seizures at 90 min after birth. There was also repetitive tiny clonic movements at the limb extremities with a transitory increase in the muscular tone. The administration of phenobarbital was partially efficacious. A second capillary hemogasanalysis revealed a significant reduction of the metabolic acidosis (pH: 7.24, PO_2_: 101 mmHg, HCO^−^_3_: 11 mmol/L, BE – 13 mmol/L), which gradually normalized within four hours.

However, severe axial muscle hypotonia with increased limb extensor muscles tone gradually became evident. The absence of crying and archaic reflexes persisted and epileptic tonic or clonic seizure started.

At 12 h of life, despite a continuous i.v. infusion of 8% glucose solution providing 7 g/kg/day of glucose, a mild hypoglycaemia (30 mg/dl) was detected; this necessitated a shift to an intake of a 10% glucose solution of 9 g/kg/day, to restore normoglycaemia. There was a transient moderate increase in his renal function parameters, most likely due to hypovolemic shock. Blood ammonia was at the upper level limit (150 μg/dL) for age, while inflammatory and metabolic markers (including lactate) were within the normal range.

On the first day of life, neonatal seizures recurred with several episodes of cyanosis associated to generalized tonic or clonic seizures, which were interrupted by increasing the Phenobarbital dose. However, the dystonic-dyskinetic clinical picture remained unchanged.

Brain ultrasonography (US) at 36 h showed diffuse hyperechogenicity, consistent with cerebral oedema; electroencephalography (EEG) tracing recorded low voltage activity with slow and wide triphasic complexes at regular intervals, which then modified to low voltage, delta-subdelta activity with wavelets in the anterior regions.

There was a strong suspicion of the presence of an inherited metabolic disease, by the first week of the child’s life, due to the persistent clinical signs of severe neurological damage i.e. stuporous state, muscle dystonia and recurrent myoclonic jerks that could not be correlated to any detectable septic parameter. Therefore, plasma amino-acids, carnitine and acylcarnitines, urinary organic acids and CSF neurotransmitters were assessed.

Concomitant brain Magnetic Resonance Imaging (MRI) scan revealed the absence of a normal high intensity signal of the posterior limb of the internal capsule on T1 weighted images and an increased basal ganglia and thalamus signal on T2 weighted images. Both findings were consistent with an hypoxic-anoxic brain damage.

Glutaric Aciduria type I (GA-I, OMIM 231670) [2, 3] was then diagnosed on the child’s 10th day of life, on the basis of elevated urinary glutaric and 3-hydroxyglutaric acid with increased glutaryl-carnitine, free carnitine depletion together with increased plasma lysine and glutaric acid levels. GA-I was then confirmed when a reduced Glutaryl-CoA dehydrogenase (GCDH, EC 1.3.8.6) activity [4] in the leukocytes (0.09 microM/h/g protein; n.v. 4.3 ± 1.8) was observed. GCDH gene mutation analysis, carried out on DNA extracted from blood lymphocytes of the proband, allowed for the identification of two mutations (p.R355H and p.R386Q) [5]. Analysis carried out on the DNA extracted from the blood lymphocytes of both parents confirmed the biparental inheritance.

It is well known that late treatment of cases with severe neurological damage at GA-I diagnosis does not allow for significant improvement in either prognosis or the clinical condition [2]. Therefore, in an attempt to interrupt the progression of the biochemical brain intoxication, an early specific low lysine diet was started, using a human milk supply and a special lysine-free and tryptophan-reduced infant formula [6, 7], supplemented with riboflavin (50 mg/day) [8] and L-carnitine (100 mg/kg/day) [9, 10].

**Fig. 1:**
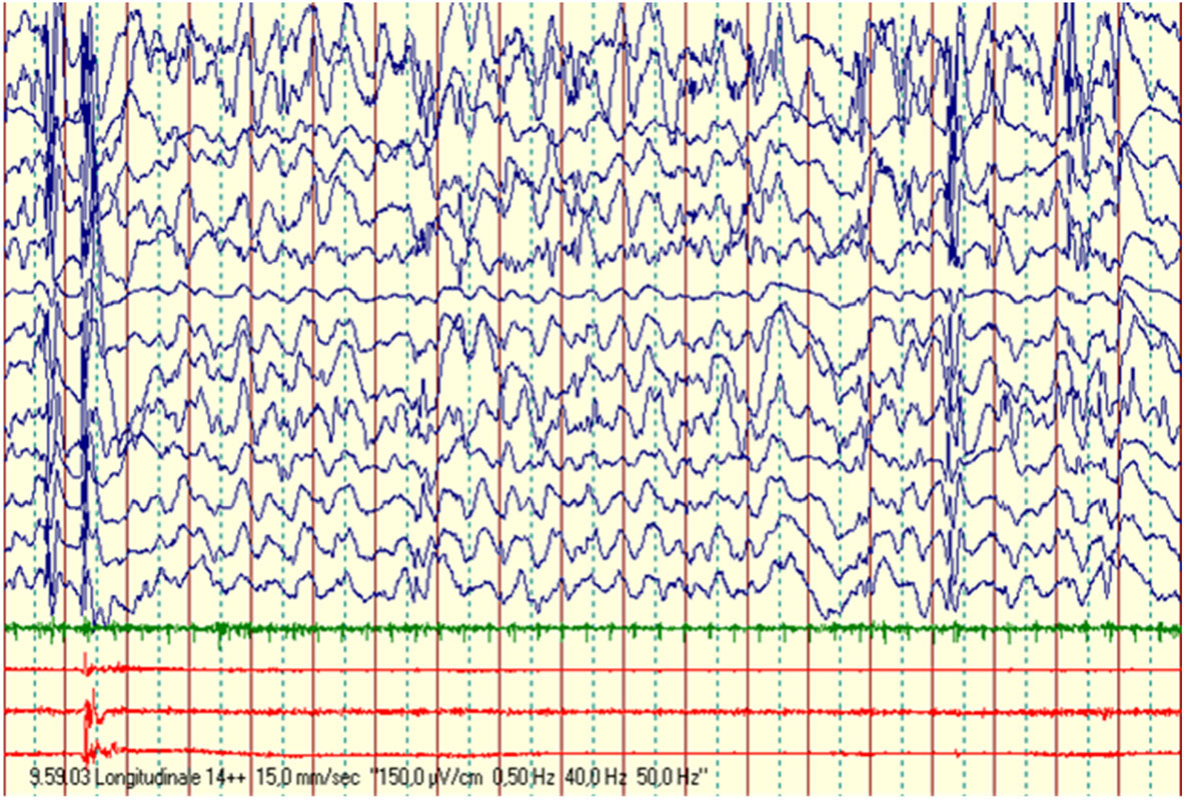
Eight months and 1 week of age, awake: global severe disorganization of background activity, with prevalent anterior bilateral high voltages slow waves, intermingled with bilateral high voltage polyspike complexes, inconstantly related to proximal limbs myoclonic jerks

As foreseen, a good metabolic control was achieved. However, there was no substantial improvement in the dystonic-dyskinetic picture. Moreover, the baby developed a severe encephalopathy with drug resistant epileptic seizures (Fig. 1).

**Fig. 2:**
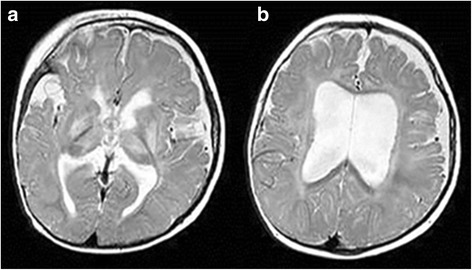
Brain MRI at one month of age. Axial T2-weighted MR image obtained at a ganglionic (**a**) and supraganglionic (**b**) level shows an increased signal intensity in the lentiform nuclei and ventrolateral thalami, in the periventricular white matter and within the subcortical U-fibers. Furthermore, the images show an enlargement of the lateral ventricles with irregular margins, fluid collection in the fronto-parietal area and a widening of the Silvian fissure

Brain MRI, at one month after birth, showed a slight reduction in the abnormal signal of the thalamic nuclei and basal ganglia together with an apparent volume loss; an enlargement of the subarachnoid cisternal spaces, a widening of the Silvian fissure with poor operculization accompanied by an expansion of pericerebral fluid spaces anterior to the temporal lobes as well as ventriculomegaly. Literature has reported such findings as being suggestive of GA-I cases with serious neurological damage [11, 12] (Fig. 2).

Attempts were made to control the seizures and improve the muscular dystonia by the administration of various anti-convulsant drug associations including: Phenobarbital, Baclofen, Clonazepam, Vigabatrin, ACTH, Clobazam, Levetiracetam, Promazine, L-dopa, Lamotrigine and Lorazepam. However, in agreement with the literature reports [13, 14] none of the therapies were efficacious.

The child required enteral (percutaneous gastrostomy) and then parenteral nutrition throughout the first year of his life, due to poor feeding and a chronic intestinal pseudo-obstruction syndrome with incoercible vomiting, unrelated with GA-I, which ensured adequate growth and metabolic control. However, neurological damage progression and a CVC-related sepsis led to exitus when the child was 2 years old.

## Discussion and conclusions

As it is well known, GA-I is a rare autosomal recessive disorder, caused by GCDH gene mutations that, in turn, lead to GCDH deficiency and accumulation of glutaric, glutaconic and 3-hydroxyglutaric acids, associated with high dicarboxylic acids concentrations and secondary deficiency of free carnitine. This metabolic profile leads to severe damage to the basal ganglia and subsequent neurodegeneration. Major clinical and neuroradiological signs are: macrocephaly, severe dystonic-dyskynetic syndrome with progressive coreoatetosis associated to frontal-temporal cerebral atrophy, retarded myelination, seizures [15], chronic subdural haematomas and degeneration of the basal ganglia, in particular of the striatum, caudate and putamen nuclei [16]. The age of clinical onset ranges from early infancy to adulthood and the severity of symptoms may vary from an acute encephalopathy with poor prognosis in early onset, to mild forms in late onset. Acute metabolic crises in asymptomatic patients may be triggered by febrile illnesses, trauma, hunger, high-protein foods and/or vaccination during a vulnerable period of brain development in infancy or early childhood [17]. GA-I can be identified by NBS, whereas prenatal diagnosis of GA-I is feasible by means of enzyme analysis on cultured chorionic villi sample (CVS) or amniocytes or by metabolite screening in amniotic fluid (AF), once an index case in the family has been identified. Molecular analysis on DNA extracted from CVS or AF is possible only when GCDH gene mutations of the index case have been previously characterized. To the best of our knowledge, the case herein described is the most precocious diagnosis of non-NBS related GA-I reported in literature to date [18]. Hypoxia and the metabolic acidosis due to complicated labour may have triggered and amplified the occurrence of metabolic decompensation in our case. GA-I worsens during stresses and/or catabolic episodes, such as PA. Moreover, the enhanced endogenous catabolism of proteins is an important route for glutaric acid production. This, along with the carnitine depletion secondary to the metabolic acidosis, led to the early onset and progression of the severe neurological damage and related symptoms. Indeed, although GA-I patients may mimic the clinical symptoms of cerebral palsy [19], they have not been reported to be at higher risk of PA. As it is known, the early onset forms of GA-I are commonly associated to the worst neurological prognosis [20]. We believe that our data evidence the importance of pre-symptomatic early diagnosis and support the opinion that a universal dissemination of an expanded NBS programme [21, 22] should be encouraged to prevent the severe neurological damage secondary to metabolic crises in early infancy and to guarantee normal and healthy lives to the majority of pre-symptomatic newborn babies with GA-I. Moreover, early and appropriate dietary lifelong treatment has to be provided [23, 24]. Nevertheless, regardless of the availability of an NBS test for the diagnosis of GA-I and other inherited metabolic diseases, it is strongly advisable that neonatologists be continuously trained to enhance their expertise in the field of differential diagnosis of neonatal neurological dysfunctions. Indeed, early diagnosis may well prevent the progression of the metabolic unbalance that underlies the brain damage, which, in turn, may reduce the risk of a poor neurological prognosis [25, 26].

## Abbreviations

AF: Amniotic Fluid
CSF: Cerebrospinal fluid
CVC: Central Venous Catheter
CVS: Chorionic Villi Sample
EEG: Electroencephalography
GA-I: Glutaric Aciduria type I
GCDH: Glutaryl-CoA-Dehydrogenase
MRI: Magnetic Resonance Imaging
NBS: Newborn screening
PA: Perinatal Asphyxia
US: Ultrasonography
